# Evaluation planning for the timed and targeted care for families program in Eastern Visayas, Philippines

**DOI:** 10.3389/fpubh.2025.1594388

**Published:** 2025-07-09

**Authors:** Yunhee Kang, Sherlyn Mae Provido, Heunghee Kim, Hee Sun Kim, Heyeon Ji, Jihwan Jeon

**Affiliations:** ^1^Department of International Health, Johns Hopkins Bloomberg School of Public Health, Baltimore, MD, United States; ^2^Department of Food and Nutrition, Seoul National University, Seoul, Republic of Korea; ^3^Research Institute of Human Ecology, Seoul National University, Seoul, Republic of Korea; ^4^World Vision Korea, Seoul, Republic of Korea

**Keywords:** timed and targeted care for families (ttCF), maternal health, child health, process evaluation, impact evaluation, Philippines

## Abstract

**Introduction:**

Timed and targeted care for families (ttCF) is a community-based intervention implemented under the Maternal, Newborn, and Child Health (MNCH) project (2021–2025) in Eastern Visayas, Philippines. The ttCF aims to improve maternal and child health during the first 1,000 days of life. The study presents the design of impact and implementation process evaluations of the ttCF program.

**Methods:**

A comprehensive evaluation framework was developed, including (1) a quasi-experimental study with 6 intervention and 6 comparison municipalities, (2) multiple cross-sectional surveys only in 16 intervention municipalities, and (3) a process evaluation guided by the RE-AIM framework (reach, impact/outcomes, adoption, implementation and maintenance). In intervention areas, barangay health workers (BHWs) conducted 12 structured home visits from pregnancy through the child’s second birthday. Comparison areas received standard government health services. The outcomes include continued health and nutrition during the first 1,000 days of life, and the utilization of antenatal and postnatal health services. Data sources include household surveys, monitoring records, and qualitative data collection.

**Results:**

In the quasi-experimental study, 1,518 pregnant or mothers with children under 2 years were enrolled, with 1,313 followed up 1 year later. For the cross-sectional surveys, 720 women were assessed at baseline and 896 at midline. As of December 2024, over 6,280 women were registered in the monitoring database. A total of 61 participants, including mothers, husbands, family members, and BHWs, were assessed at focus group discussions and in-depth interviews.

**Discussion:**

The comprehensive evaluation will provide an in-depth understanding of the ttCF impact pathway, strategic monitoring, and the quality of program implementation. The evidence will inform the strengthening of community health system by promoting BHW engagement and guiding community interventions to improve maternal and child health outcomes in the Philippines.

## 1 Introduction

Between 1990 and 2023, a remarkable decline in child and maternal mortality was reported globally. Under-five mortality fell from 13.0 million to 4.8 million (1) while maternal mortality dropped from 443,000 deaths (328 per 100,000 live births) in 2000 to 260,000 deaths (197 per 100,000 live births) in 2023 (2). These improvements were driven by increased investments in reproductive, maternal, newborn, and child health and nutrition (RMNCH), the scale-up of evidence-based interventions, and improvements in broader socio-economic determinants (3–5). Yet, despite significant gains, current approaches remain insufficient to address effectively the growing burden of maternal health and well-being throughout the life course (6, 7).

Community health workers (CHW) system can fill the gap between the high accessibility of antenatal care services and poor maternal and child health outcomes in the region, through a translation of health services and guidance on healthy behaviors within a cultural context. Complied evidence supports that CHW has been effective in frontline primary health care management in improving mother and child health. A meta-analysis of RCTs in Bangladesh, India, Nepal, and Malawi reported a 23% reduction in neonatal mortality and a 37% reduction in maternal mortality among women’s groups with participatory learning and action (8).

In the Philippines, maternal mortality has reduced notably by 35% from 127 to 84 deaths per 100,000 live births over the past two decades (2). The fertility rate also marked a decline during the same period, from 3.7 births in 2000 to 1.9 births per woman in 2023 (9). However, disparities in maternal and child health within the country persist, calling for holistic interventions to support marginalized populations (10, 11).

Eastern Visayas is a geographically isolated and disadvantaged area (12). According to the 2022 Demographic and Health Survey, the neonatal mortality rate was 13 deaths and under-five child mortality rate was 27 deaths per 1,000 live births in the Eastern Visayas (13). Moreover, the poverty rate in Eastern Visayas in 2018 was 49.5 percent, significantly higher than the national rate of 16.7 percent. In 2015 National Nutrition Survey (14), Eastern Visayas ranked in second place regarding malnutrition status, where 41.7% of children under 5 years were stunted, 8.4% wasted, and 29.5% underweight.

Barangay health workers (BHWs) serve as front-line workers, particularly critical roles in rural areas (15). BHWs deliver and support services related to maternal and child health, integrated community case management, HIV/AIDS, malaria, and tuberculosis control and implement national nutrition programs throughout the continuum of care. However, the impact of BHW programs on health outcomes is limited, likely due to limited training, resources, and a lack of community support (16).

### 1.1 Intervention

#### 1.1.1 Maternal, newborn, and child health (MNCH) project

**Figure 1:**
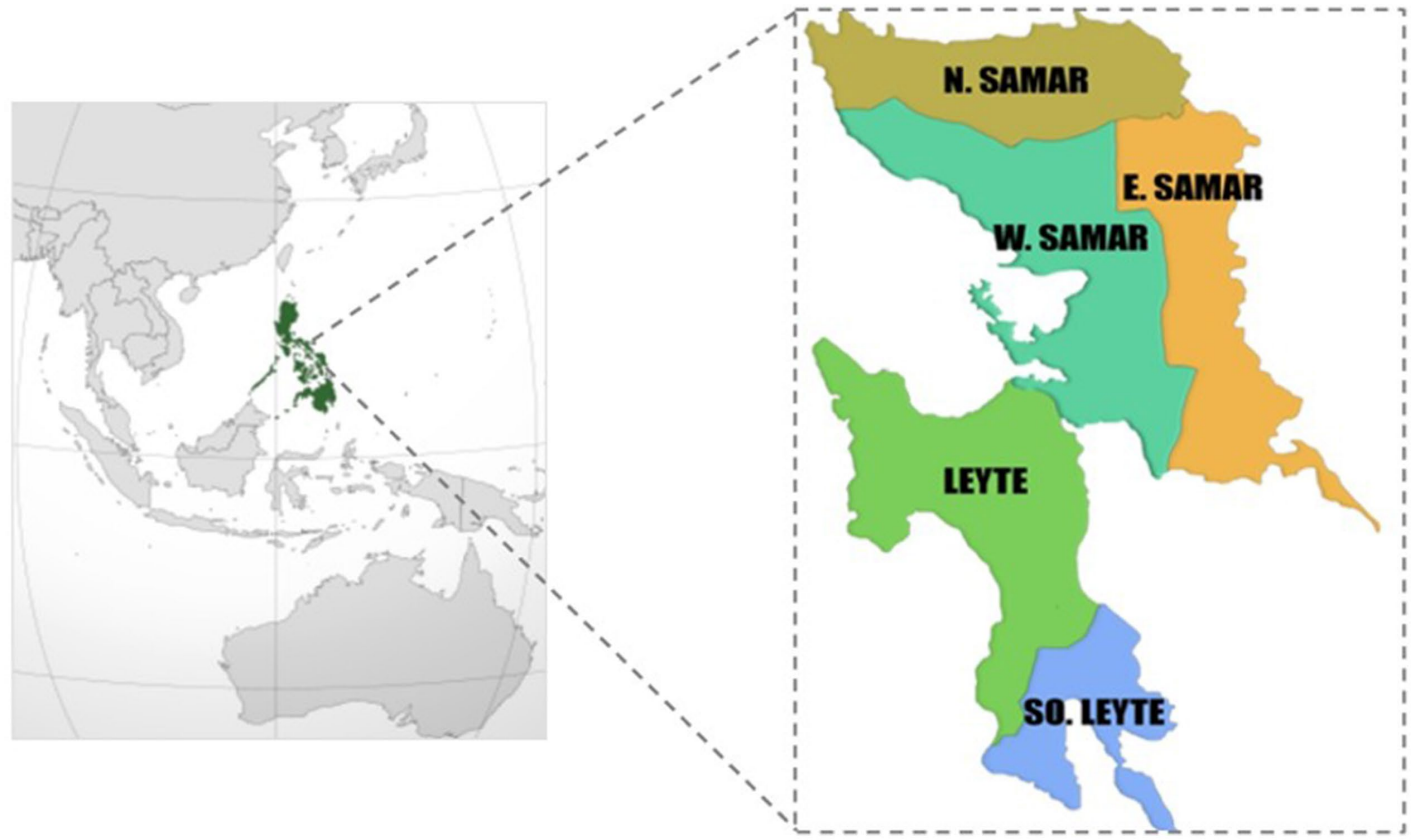
Timeline of household visits by barangay health workers from a timed and targeted care for families (ttCF) program (adopted from World Vision Korea).

The maternal, newborn, and child health (MNCH) project (2021–2025) was adopted as part of KOICA’s strategic partnership program, which attempted to harness the expertise and grassroots networks of Non-Governmental Organizations over 15 years. The MNCH project targets vulnerable households including Geographically Isolated and Disadvantaged Area (GIDA) in the provinces of West Samar, Northern Samar, East Samar, and Leyte provinces (Figure 1). Through active community engagement and decision-making involvement, the MNCH project aims to enhance access to and utilization of health services in these areas. To ensure the delivery of quality MNCH services at both facility and household levels, World Vision equipped health professionals and barangay health workers. Simultaneously, it has enhanced the availability and readiness of health facilities by reinforcing the referral system and providing essential equipment, medicines, and consumables.

### 1.2 Timed and targeted care for family (ttCF)

The timed and targeted care for family (hereafter, ttCF) approach, a key MNCH component, adapts the Timed and Targeted Counselling (ttC) model by incorporating local contextual factors and emphasizing family participation and gender equality to improve maternal and child health during the first 1,000 days. The ttC is World Vision’s (WV) flagship program for maternal and child health emphasizing a family-inclusive and gender-transformative approach through the assistance of trained community-based health workers (CHWs) (17). ttC programs have been conducted in 38 different countries around the world (18). ttC programs have demonstrated a wider range of outcomes across the continuum of care including improved antenatal care and newborn health care, increased facility delivery, greater family planning uptake, enhanced breastfeeding and complementary feeding behaviors, hygiene practices, and child nutritional status (19–22). As ttC visits are conducted by community health workers or volunteers, the program strengthens access to primary health care and reinforces the community health system (23). According to the WHO Macroeconomics in Health Commission, an intervention is considered cost-effective if the cost per discounted life-year saved is below the national GDP per capita. Based on this criterion, ttC programs have been shown to be cost-effective (31). A ttCF monitoring tool was created through a collaborative effort across different sector experts from World Vision. The two key components of monitoring system are household registration and the development of a centralized database. For the register, a nine-page booklet was initially developed by World Vision International in 2015 and later modified by a partnership of the MNCH project team and the Department of Health (DOH)-Philippines for the contextualization of the tool. After extensive consultation with various stakeholders, the materials were printed in the second quarter of 2023.

The ttCF in the Philippines introduces several key adaptations compared to the previous ttC model. Not only barangay health workers (BHW) but also Barangay Nutrition Scholars (BNS) are also engaged in home visit counseling, expanding the reach of community-based health services. The Department of Health has also begun implementing the minimum requirement of eight antenatal care (ANC) visits, in line with WHO guidelines. This recommendation is incorporated into the CHW’s messaging, aiming to promote healthier pregnancies through the prevention, early detection, aiming to promote healthier pregnancies through the prevention, early detection, and referral of complications, while also encouraging enrollment in Phil health, the national health insurance program, to ensure financial access to these essential services. Under current DoH guidelines, a postnatal visit is defined as occurring within 45 days after delivery, extending the previous standard of 42 days to allow better follow-up and support. Furthermore, Shaken Baby Syndrome is indicated as a form of child abuse, highlighting the need for caregiver education and early prevention. The ttCF manual assigns specific roles to male caregivers and partners, which were not included in the previous ttC program.

The ttCF program trained BHWs and BNSs, supervised by the Rural Health Unit (RHU), to strengthen the capacity to support families in understanding the key maternal health practices from pregnancy to 2 years of life. In 2022, a total of 42 healthcare professionals (comprising doctors, nurses, and midwives) from 16 municipalities and three provincial health officers were trained with the ttCF training of facilitators (ToF) curriculum. In April–June 2023, these previously trained health workers trained 1,865 BHWs (female: 1,826; male: 39) for ttCF messages and counseling skills in their respective areas. Pregnant women and postpartum mothers with children 0–23 months of age have been visited by trained barangay health workers (BHWs) since July 2023. BHWs were provided with non-monetary compensation for household visits.

The ttCF database serves as the primary monitoring tool of the project by enabling a robust analysis of the collected data. As of December 2024, the database contains 6,280 ttCF households with pregnant and lactating women with infants 0–2 years old. The register is presented by BHWs during household visits, and the data is subsequently reported to RHU supervisors (e.g., rural health manager or nurses) then to each field facilitator to ensure minimal error prior to handing over the encoder. If there is missing data, the encoder returns the register to the field facilitators. If a common data issue is found in the registers, the field facilitator or program officer advises the BHWs to revisit the households and collect missing data. M&E officers validate the data and share the summary data with the research team.

## 2 Methods

### 2.1 Study purpose

**Figure 2:**
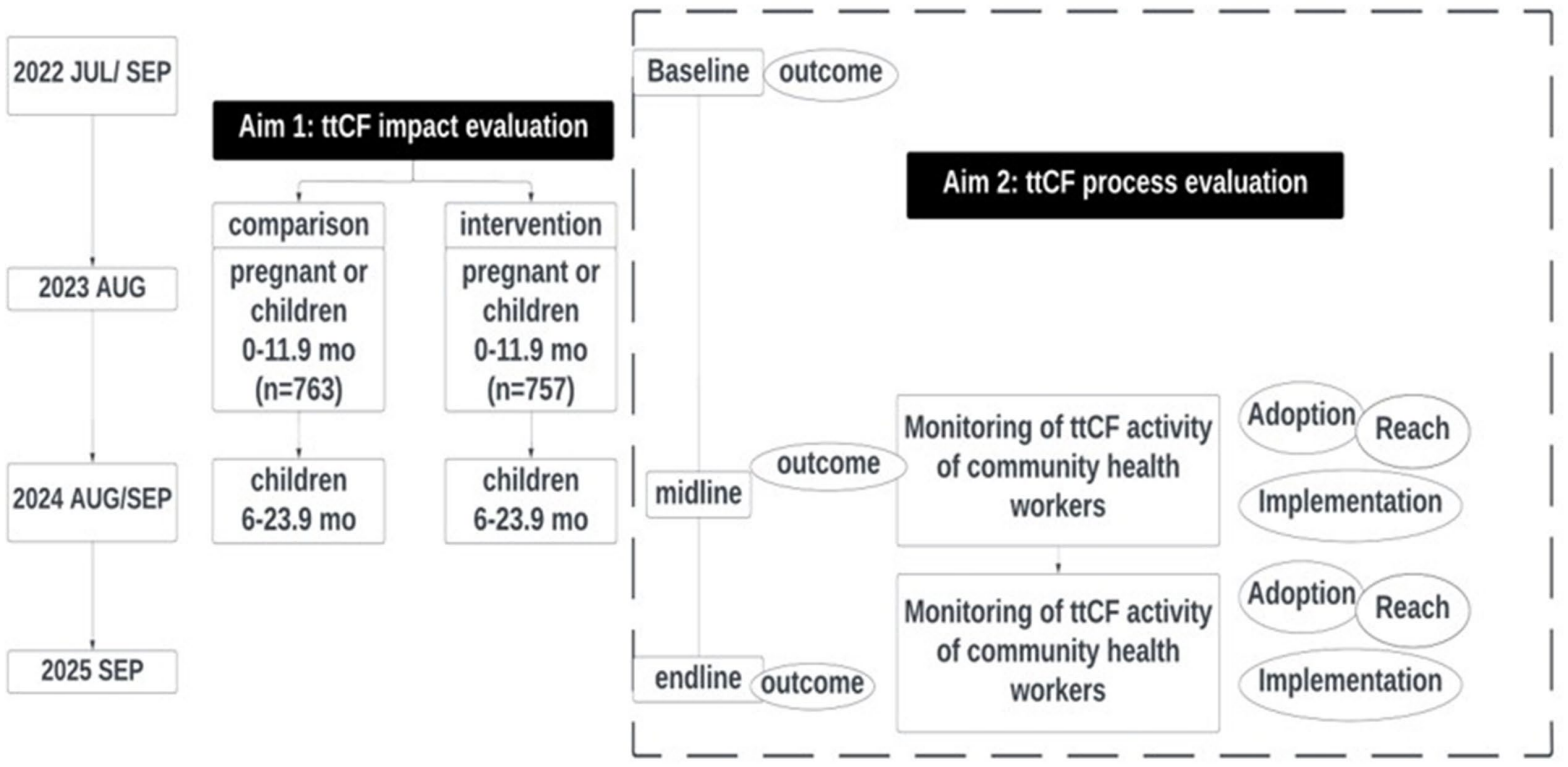
Monitoring and evaluation framework of a timed and targeted care for families (ttCF) program.

The present study aims to describe the impact and implementation process of ttCF program on maternal and child health and nutrition outcomes and service utilization. To understand the impact and process of ttCF program in Eastern Visayas, Philippines, a comprehensive evaluation was planned with the following objectives: (1) to examine the impact of ttCF on utilization of maternal and child health services and continued maternal and child health and nutrition outcomes and (2) to conduct a process evaluation of the ttCF program using the RE-AIM evaluation framework: (1) reach; (2) impact/outcomes; (3) adoption; (4) implementation; and (5) sustainability. The evaluation framework is described in Figure 2. The research team, MNCH staff, and community health workers collaborate to establish the M&E system and collect various data.

### 2.2 Impact evaluation

#### 2.2.1 Study areas

The study applies a quasi-experimental design in 12 municipalities, following pregnant women or mothers aged 15–49 years old with at least one child aged 0 to 11.9 months. Selection of 12 study municipalities was made through discussions with research team, MNCH team, and local governments. Out of the four MNCH project provinces, only West Samar and East Samar were included in the study due to their larger expected population size. Northern Samar province was excluded too far from the MNCH office in Tacloban where research management is made and are residing within West and East Samar provinces of Eastern Visayas. Out of 12 study municipalities, 6 municipalities of Taft, General MacArthur, Quinapondan from East Samar and Marabut, Basey, and San Jorge from West Samar were assigned intervention areas and the remaining 6 municipalities of Giporlos, Oras, and San Julian from East Samar and Pinabacdao, Hinabangan, and San Sebastian from West Samar were assigned as comparison areas.

#### 2.2.2 Intervention

**Figure 3:**
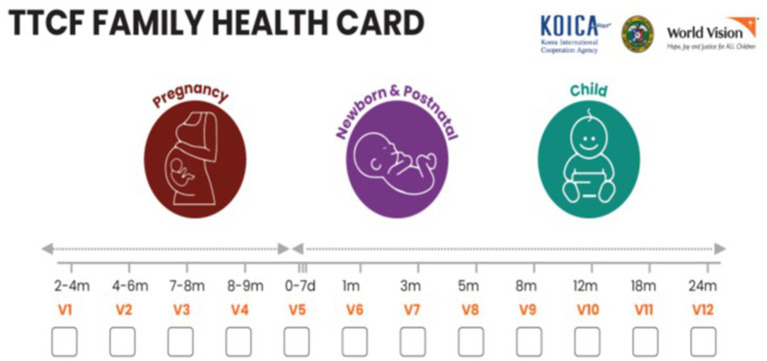
Study areas (adopted from World Vision Korea).

For the participants in the intervention group, 12 home visits are scheduled according to the manual, with 4 visits during pregnancy, 4 visits from birth to 6 months of child age, and 4 visits between 6 to 24 months of age. Twelve home visits are scheduled in total: during pregnancy (4 visits), from birth to 6 months of age (4 visits), and thereafter to 24 months after birth (4 visits) (Figure 3). The messages include a wide range of health and nutrition topics related to pregnancy, delivery, postnatal care, and early childhood childcare. These include tracking of pregnant women and children, WASH, breastfeeding, identification of danger signs, and referral (Supplementary Table 1). Health messages and counseling are delivered through storytelling techniques supported by visual materials. Through the use of visual materials and sharing of cases in the form of stories, questions are asked to discuss together what positive behaviors mothers and guardians are currently engaging in and what needs improvement. BHWs document the health practices observed and done by each household. For health practices not done by the household, negotiated agreements are established and recorded. In comparison areas, the government’s routine antenatal and postnatal health services are provided. Household visits by BHWs are practiced but not promoted by the government.

Prior to the study enrollment, the research team obtained the lists of eligible pregnant women or postpartum women from BHWs, RHUs, and the MNCH project team. Pregnant or postpartum mothers with children aged 0–11.9 months are eligible for enrolment. Enumerators will assess eligibility and obtain informed consent through household visits. If two or more women in a household meet the eligibility criteria, the enumerator will randomly choose one. If there are multiple children within a household, the youngest child will be selected for the study.

#### 2.2.3 Sample size

The sample size of 760 per group was necessary to test the 8% difference in the minimum dietary diversity (MDD) of children aged 6–23 months after 1 year of follow-up, with consideration of 15% loss to follow-up and 1.2 design effect (24). To reach this number we aim to recruit an average of 127 participants from each of the 12 municipalities. A planned sample of 232 pregnant women will allow detection of a 10% difference in antenatal care visit coverage with 80% power and 5% significance.

#### 2.2.4 Outcomes

Maternal-level outcome variables include antenatal care, facility-based delivery, and postnatal visits, including (1) 8 or more antennal visits (a new recommendation by the Philippine government), (2) dietary diversity, (3) timely postnatal care, (4) nutritional status (e.g., BMI), and (5) mental health. At the child level, outcome variables of focus will include (1) exclusive breastfeeding and continued breastfeeding, (2) minimum dietary diversity, and (3) prevalence of stunting (LAZ < -2). Background information on the individual (e.g., literacy, age, pregnancy status); household characteristics (e.g., size, residence location); prenatal, childbirth and postpartum care services; child nutrition and health status; and home visit counseling service will also be collected so that these can be controlled for or used for disaggregation in data analysis.

### 2.3 Cross-sectional surveys

Multiple cross-sectional surveys have been conducted in 16 MNCH municipalities of all four provinces. The cross-sectional data will track the changes in health outcomes at the population level over the MNCH project period (2022, 2024, and 2025). Lot Quality Assurance Sampling (LQAS) was used to determine the sample size of the cross-sectional surveys. LQAS is used to assess a program by analyzing data produced by a small sample. LQAS recommends using 19 supervision areas (SA) as the primary data collection unit. In this survey, 19 barangays were randomly selected in each province. Barangays were selected using the Probability Proportional to Size (PPS) sampling method. The sample size of *n* = 19 and decision rule of *d* = 13 was selected to maintain *α* error of ≤10%, and *β* error of ≤10%. It is interpreted that 80% coverage is achieved when more than 13 positive responses out of 19 responses are recorded. In practice, on average, 10 households were surveyed per barangay, based on field feasibility. Starting from the first household, the enumerator moved in a predetermined direction, visiting households until eligible respondents from each target group were found. In barangays with fewer than 10 eligible households, enumerators surveyed adjacent barangays after consultation with a survey supervisor. In each province, the number of households assessed was 190. The midline and the endline surveys will assess trends of antenatal, delivery, and postnatal care from baseline, midline to endline. The same barangays will be revisited for the midline and endline surveys, with an adjusted sample size to enable trend analysis over time. Assuming that the proportion of 8 or more antenatal checkups (a recent recommendation by the Philippines government) at the baseline was 15.6%, we expect a total increase of 8% in the outcome for 4 years, a 2% annual increase from the baseline to the endline with significance level 5%, design effect of 2, and non-response rate of 10%. The minimum required sample size is 900 participants. Thus, 225 participants are included in each of the four provinces, and 56 participants in each of the 16 municipalities. Each Barangay is divided into segments containing a roughly equal number of households; a segment is then chosen at random. This process is repeated until approximately 11 to 12 eligible households are identified in each barangay.

### 2.4 Process evaluation

Most evaluation studies have focused on the impact of ttC on improvements in key health indicators considered from conception to 2 years of age. However, most ttC evaluation studies lack information on the implementation process. For example, the ttCF model schedules 12 home visits by BHWs over a period of nearly 3 years (1,000 days) per household. Process evaluation of such a long-term intervention requires substantial time and effort to understand the program pathways. Currently, there is insufficient evidence to determine whether these home visits take place as scheduled and in the intended number. In addition, household visits by community health workers are not mandatory and are driven by limited incentives, without additional compensation. In South Africa, the coverage of household visits by CHW was not as high as 17%, and one to two households were visited per day per CHW (25). A process evaluation in Ethiopia of monthly household visits to pregnant and children under 2 years of age reported that only 28.2% of intervention mothers had a visit by community volunteers for nutrition counseling in the past 3 months (32). For counseling by CHWs to be effective, not only must project participants create health behavior changes for pregnancy, childbirth, and childcare, but also community health facilities must provide relevant quality services. These challenges may affect the sustainability of the ttCF program.

The process evaluation will characterize the process implementation of the ttCF program using the RE-AIM planning and evaluation framework: (1) reach; (2) impact/outcomes; (3) adoption; (4) implementation; and (5) maintenance. Since its development in 1995, the RE-AIM framework has been widely used to design and evaluate community-based health promotion programs. RE-AIM framework is one of the most used planning and evaluation frameworks across the fields of public health, behavioral science, and implementation science (26).

Process evaluation study using the RE-AIM framework allows the utilization of various data sources produced during the MNCH program implementation (27). Our process evaluation attempts to reveal the program impact pathways and factors that may explain the impact of the ttCF program on maternal and child health outcomes. Multiple data sources will be used to assess the process evaluation components including a quasi-experimental study, multiple cross-sectional surveys, monitoring and performance records, and qualitative data collection. World Vision Philippines/World Vision Korea will prepare annual and semi-annual performance reports for internal review and grant reporting. Those reports will be used to extract the relevant data by the research team. World Vision’s Excel-based ttCF monitoring form has been used in several countries globally. Table 1 describes the key outcomes for each construct of the RE-AIM framework.

**Table 1:** The overview of RE-AIM framework components to conduct timed and targeted care for families (ttCF) process evaluation.

Components	Example of evaluation questions	Eligibility	Methodology
Reach (Percent of participants among the eligible population)	Percentage of pregnant women enrolled in ttCF program among eligible mothers	Pregnant women or women with one or more children aged 0–23 months in 16 project municipalities	ttCF Monitoring records World Vision’s performance Report Records from Rural Health Unit (RHU)
Impact (Impact/effectiveness at the individual level)	Percentage of access to postpartum care services Percentage of Exclusive breastfeeding Percentage of Minimum dietary diversity among children 6–23 months of age	Pregnant women and mothers of children aged 0–23 months in 16 project municipalities	Secondary data of baseline survey (2022) Midline (2024) and Endline (2025) Survey Cohort surveys (2023–2024)
Adoption (Application rate of Project activities)	Percentage of BHW who actually performed ttCF home visits out of all community health workers in the project area	~2000 BHW in 16 project municipalities	ttCF Monitoring record World Vision’s performance Report Qualitative study (2024)
Implementation (The extent to which the project was actually executed compared to planning)	Number of households where BHW conducted home visit counseling Number of BHW who completed ttCF training	~2000 BHW in 16 project municipalities	World Vision’s Performance Report ttCF Monitoring records
Sustainability: (The extent to which Project activities continue)	Percentage of mothers who are satisfied with BHW’s counseling performance	Pregnant women and mothers of children aged 0–23 months in 16 project municipalities	Midline (2024) and Endline (2025) Survey Cohort surveys (2023–2024) Qualitative study (2024)

#### 2.4.1 Reach (at the individual level)

Reach (at the individual level) is defined as the number or percentage of individuals enrolled in interventions out of the eligible population. As a measure of the “reach” component, the proportion of pregnant women who are enrolled in the ttCF program will be calculated for each of the 16 project municipalities, using the number of pregnant women extracted from the monitoring tool. The proportion will be calculated in percentages for each province or municipality. The data sources for “reach” component are monitoring records, performance reports, and records from RHUs.

#### 2.4.2 Impact/outcomes

The “impact/outcomes” component will be measured mainly using cohort follow-up surveys and multiple cross-sectional surveys. The multiple cross-sectional surveys will be conducted 16 MNCH municipalities, 4 provinces (Northern, West, and East Samar, and Leyte) while the cohort survey will be conducted in six intervention and six comparison municipalities in two provinces (East and West Samar). The cross-sectional data will track the changes in population-level health outcomes over a four-year study period (2022–2025) and the cohort study will measure 1 year of program impact following the same study participants. The impact indicators of ttCF are antenatal, delivery, and postnatal service utilization and health and nutrition indicators among pregnant and lactating women and children 0–24 months of age. Each indicator will be measured in percentages or numbers and will be summarized for each province and municipality.

#### 2.4.3 Adoption

Adoption is defined as the extent to which project activities are implemented by BHWs. The adoption components are mainly evaluated among ~2000 BHWs in 16 project municipalities to examine to what extent BHWs performed household visits according to the guidelines. The main indicators are the percentage of BHW who performed home visits out of all community health workers in the project area. Data will be extracted from monitoring records and performance reports.

#### 2.4.4 Implementation

Implementation is defined as the extent to which the project was conducted compared to planning. The ttCF implementation will be assessed at the stage of training and household visits among ~2000 BHWs. According to the ttCF guidelines, pregnant mothers are supposed to have four visits, at delivery to 6 months of age four visits, and 6–23 months of age another four visits. We calculate the percentage of BHWs who completed ttCF training out of all BHWs. We are interested in the percentage of households with ever home visit counseling by BHW, households with timely visits and messages at pregnancy, postnatal, and up to 23 months of age. In addition to monitoring records, study participants at cohort and cross-sectional surveys will be asked how many times they had home visit counseling by BHWs since their latest pregnancy. The data will be presented by province and by project municipality, and further investigation is made if the dose delivered of ttCF at each municipality will be associated with health outcomes.

#### 2.4.5 Sustainability

Sustainability is defined as the extent to which program activities continue. Study mothers will be asked for their satisfaction with ttCF visits, and self-efficacy in putting the messages into practice. A qualitative study will determine the facilitators and barriers to ttCF activities, service satisfaction, and areas requiring improvement. This study plans to conduct in-depth interviews (IDI) and focus group discussions (FGD) with mothers, family members and BHWs.

## 3 Results

### 3.1 Quasi-experimental study

Study enrollment took place in September 2023, with 763 mothers in comparison areas and 755 in intervention areas. Among them, 470 were pregnant, 1,046 were postpartum women, and four women were pregnant and had a baby under 12 months of age. After a year, 1,313 (655 in comparison areas and 658 in intervention areas) were followed up in September 2024. The intention-to-treat principles will be applied in data analysis. The comparability of sociodemographic and demographic characteristics between the intervention and comparison groups will be assessed using Chi-square tests or *Student’s t-*tests. Generalized linear models (GLM) will be used to estimate the program impact on child diet and nutritional status. The difference-in-difference (DID) approach will estimate the program impact on maternal mental health and dietary quality, adjusting for the baseline differences.

### 3.2 Cross-sectional survey

A total of 720 households across four provinces were included in the baseline survey. The baseline data collection was conducted in July 2022 in East Samar/Leyte and in October 2022 in Northern/West Samar. The midline study was conducted in September 2024 (Table 1) and the endline study is scheduled for September 2025.

Exploratory data analysis will be conducted to calculate mean (SD) and percentages for all variables collected during different surveys. Descriptive statistics will be stratified by province (East Samar, West Samar, Leyte, and North Samar) and comparability across the four provinces will be tested using *χ*^2^ tests for categorical variables and Student’s *t*-tests for continuous variables. Key indicators will be selected to assess health service utilization during ANC, delivery, and PNC, family planning, and maternal and child nutritional status. These will include the proportion of women attending at least four ANC visits, receiving skilled birth attendance, obtaining postnatal care within 48 h of delivery, using contraceptives, and the prevalence of undernutrition among mothers and children under 5 years.

### 3.3 Process evaluation

We recruited 61 participants through purposive sampling with the support of MNCH project team. A semi-structured interview guide was used during in-depth interviews (IDI) and focus group interviews (FGD). In September 2024, data collection was carried out in two municipalities. In Basey municipality, one FGD with 8 mothers, four IDIs with mothers, one FGD with 7 husbands/partners and four IDIs with mother-in-law, one FGD with 7 BHWs, and four IDIs with BHWs were conducted. In Marabut municipality, we conducted two FGDs with mothers (one with 6 mothers, and another with 4 mothers), one FGD with 4 husbands/partners and four IDIs with mother-in-law, one FGD with 4 BHWs, and four IDIs with BHWs. Interviews will be transcribed verbatim and translated from Waray-Waray into English. We will conduct a thematic analysis through a collaborative review process using the MAXQDA 24 program. Initial open coding will be used to identify keywords and phrases, followed by selective coding to categorize and analyze the data into three overarching themes: enabling factors, inhibiting factors, and sustaining factors of the ttCF program.

## 4 Discussion

This study introduces the evaluation framework of ttCF program, which aims to improve maternal, newborn, and child health outcomes during the first 1,000 days of life in Eastern Visayas, Philippines. The program attempts to increase the utilization of prenatal and postpartum services and support the health of pregnant and lactating women and children under 2 years of age. A comprehensive evaluation strategy was employed including a quasi-experimental study, multiple cross-sectional surveys and a process evaluation. This paper outlines the methodological design, evaluation process, and progress to date.

Previous evaluations of BHWs in the Philippines have largely focused on qualitative studies exploring specific components of their role (16, 28, 29, 33). To our knowledge, the present study is the first large-scale evaluation in the Philippines to assess maternal and child health outcomes during the first 1,000 days of life through an integrated evaluation framework.

The collaborative monitoring and evaluation system developed by the MNCH project staff and research team has enabled a comprehensive understanding of the ttCF program implementation. This partnership facilitated rigorous data collection and analysis by research team, while allowing the project staff to interpret and apply findings in a meaningful and actionable manner. Sharing and utilizing collected data can enhance real-time program insights but also provide strategic direction for scaling-up efforts.

With 12 scheduled household visits spanning from the prenatal period to a child’s second birthday, ttCF enables finding of a trajectory of maternal and child health status over time. Continuous training, motivation, supervision, and support of BHWs are critical to a successful implementation of the ttCF program and long-term participant retention. By nature, BHWs often face competing priorities as they are engaged in multiple health programs. Thus, careful management of individual workloads and continuous social and institutional support are essential to maintaining program quality and preventing burnout.

In Eastern Visayas, the population-to-BHW ratio is reported 52:1, lower than the national average of 88:1 (13). Given the critical role that BHWs play in supporting maternal and child health and their expanding responsibilities in promoting lifecycle well-being and the prevention of non-communicable disease, it is essential to acknowledge the increasing workload. Despite limited resources and minimal compensation, BHWs are expected to carry out a wide range of health-related tasks. Findings from the current study may serve as evidence to advocate for adequate staffing, capacity building, and resource allocation for context-specific primary healthcare.

The ttCF checklist was introduced for the first time in the Philippines through the MNCH project and includes a comprehensive set of items related to antenatal, newborn care, and postnatal check items to be assessed at each home visit. Approximately 2,000 BHWs use this paper-based checklist, making quality control a significant undertaking. Most BHWs are aged 40 to 60, limiting the feasibility of mobile-based data collection. BHWs are supervised by RHU midwives, who review the paper forms before submission to the MNCH team. This process results in a time lag between data collection and data processing, potentially delaying program responses. Gradual adoption of a real-time digital monitoring system could reduce these delays, allowing faster responses to the participant’s needs and improving overall program quality and impact.

Equally important is the strategic timing of monitoring and evaluation (M&E) activities, which ensures a continuous feedback loop to guide activity adjustments. Timely access to accurate data allows for ongoing course corrections and responsive programming tailored to the evolving needs of the communities served.

Several limitations of the study should be acknowledged. During the implementation period, similar public health promotion programs were reported in parts of the comparison area, potentially influencing BHW activities. These external factors likely introduced contamination in estimating the ttCF impact. As a result, implementing a randomized controlled trial (RCT) or quasi-experimental design in real-world settings proved challenging. The study anticipates recruitment challenges in identifying pregnant women during the early stage of pregnancy. Since program impact is maximized when mothers are enrolled from early pregnancy, delayed recruitment may reduce the potential benefits of the intervention. Over 60% of enrolled participants joined with children aged 0–11.9 months, limiting exposure to the full ttCF package. Further Eastern Visayas has geographically isolated and disadvantaged areas, some intervention communities were not reached within the current study design. These limitations may affect the representativeness and generalizability of the findings across Eastern Visayas.

While most evaluation frameworks in maternal and child health programs were limited to examining the program impact, our RE-AIM framework considered a comprehensive, multi-layered process and evaluation using various data collection tools such as quantitative, monitoring records and qualitative data tools.

Since the ttCF program was the first ever introduced in the Philippines, our evaluation will determine the extent to which the program has achieved the intended health and nutrition outcomes and identify remaining gaps in reaching the targeted indicators. The mid-term impact data will inform practitioners and donors to make decisions on long-term program planning, funding, and sustainability after the program cessation. Qualitative findings can highlight individual and contextual factors that support or hinder BHW’s home visits and to what extent the newly introduced ttCF model is integrated into the existing community health system. By assessing program reach, adoption, and sustainability, our research contributes valuable evidence to implementation science in maternal and child health in Southeast Asia.

Furthermore, the ttCF program aligns with the recently adopted Magna Carta of BHWs in the Philippines, which underscores various support and capacity building for BHWs (30). Our study findings will help position the ttCF program in line with government efforts to strengthen community health systems.

## Glossary

ttCF: Timed and Targeted Care for Families
MDGs: Millennium Development Goals
RMNCH: Reproductive, Maternal, Newborn, and Child Health and Nutrition
SDGs: Sustainable Development Goals
CHW: Community Health Worker
BHW: Barangay Health Worker
WV: World Vision
MNCH: Maternal, Newborn and Child Health
KOICA: Korea International Cooperation Agency
GIDA: Geographically Isolated and Disadvantaged Area
LGU: Local Government Unit
BEmONC: Basic Emergency Obstetric and Neonatal Care
CEmONC: Comprehensive Emergency Obstetric and Newborn Care
RHU: Rural Health Unit
BNS: Barangay Nutrition Scholars (BNS)
PhilHealth: Philippine National Health Insurance
DOH: Department of Health
RE-AIM: Reach, Impact/outcomes, Adoption, Implementation, and Sustainability
MDD: Minimum Dietary Diversity
LQAS: Lot Quality Assurance Sampling
PPS: Probability Proportional to Size
IDI: In-depth interviews
FGD: Focus group discussions
EVHRDC-ERC: Eastern Visayas Health Research and Development Consortium Ethics Review Committee
JHSPH IRB: Johns Hopkins Bloomberg School of Public Health Institutional Review Board
