# Supplementary material for: Evaluation planning for the timed and targeted care for families program in Eastern Visayas, Philippines
Source: Front Public Health. 2025 Jul 9;13:1594388. doi: 10.3389/fpubh.2025.1594388 (PMC12283334; doi:10.3389/fpubh.2025.1594388)
Supplement: Supplementary file 1 [file Table_1.DOCX]

**Supplemental Table 1. Key messages of a timed and targeted care for families (ttCF) program by time of visit**

|  | Pregnancy | | | Essential Newborn and Postnatal Care | | Children 0-24 months | | | | | | |
| --- | --- | --- | --- | --- | --- | --- | --- | --- | --- | --- | --- | --- |
|  | V1 | V2 | V3 | V4 | V5 | V6 | V7 | V8 | V9 | V10 | V11 | V12 |
| Checklist | 2-4 m | 4-6 m | 7-8 m | 8-9 m | 0-7d | 1m | 3m | 5m | 8m | 12m | 18 m | 24m |
| Additional serving | v | v | v | v |  |  |  |  |  |  |  |  |
| Balanced and diverse meal | v | v | v | v |  |  |  |  |  |  |  |  |
| Iodized salt | v | v | v | v |  |  |  |  |  |  |  |  |
| Iodine capsule | v | v | v | v |  |  |  |  |  |  |  |  |
| Iron rich diet | v | v | v | v |  |  |  |  |  |  |  |  |
| Iron and folic acid | v | v | v | v |  |  |  |  |  |  |  |  |
| Calcium Carbonate |  | v | v | v |  |  |  |  |  |  |  |  |
| ANC visits | v | v | v | v |  |  |  |  |  |  |  |  |
| Blood Typing | v |  |  |  |  |  |  |  |  |  |  |  |
| Birth plan | v | v | v | v |  |  |  |  |  |  |  |  |
| Mosquito net used regularly | v | v | v | v |  |  |  |  |  |  |  |  |
| Handwashing with soap | v | v | v | v |  |  |  |  |  |  |  |  |
| Access to improved latrine | v | v | v | v |  |  |  |  |  |  |  |  |
| Deworming (once only) |  |  | v | v |  |  |  |  |  |  |  |  |
| Hemoglobin Count | v |  |  |  |  |  |  |  |  |  |  |  |
| Hematocrit Count | v |  |  |  |  |  |  |  |  |  |  |  |
| Gestational Diabetes Screening | v |  |  |  |  |  |  |  |  |  |  |  |
| Hepatitis B Screening | v |  |  |  |  |  |  |  |  |  |  |  |
| HIV testing | v |  |  |  |  |  |  |  |  |  |  |  |
| Syphilis Screening | v |  |  |  |  |  |  |  |  |  |  |  |
| Danger Signs | v | v | v | v |  |  |  |  |  |  |  |  |
| Facility birth |  |  |  |  | v |  |  |  |  |  |  |  |
| Skilled attendant |  |  |  |  | v |  |  |  |  |  |  |  |
| Family planning |  |  |  | v | v | v |  |  |  |  |  |  |
| Iron and folic acid |  |  |  | v | v | v |  |  |  |  |  |  |
| Balanced & diverse meal |  |  |  | v | v | v |  |  |  |  |  |  |
| Postnatal consultation |  |  |  | v | v | v |  |  |  |  |  |  |
| Maternal hygiene |  |  |  | v | v | v |  |  |  |  |  |  |
| Water stored safe & ready |  |  |  | v | v | v |  |  |  |  |  |  |
| Access to improved latrine |  |  |  | v | v | v |  |  |  |  |  |  |
| Rest & family support |  |  |  | v | v | v |  |  |  |  |  |  |
| Partner/family participation |  |  |  | v | v | v |  |  |  |  |  |  |
| Danger signs |  |  |  | v | v | v |  |  |  |  |  |  |
| Calcium Carbonate |  |  |  | v | v | v |  |  |  |  |  |  |
| Essential newborn care |  |  |  |  | v | v |  |  |  |  |  |  |
| Care of the cord |  |  |  |  | v |  |  |  |  |  |  |  |
| Vaccination (Birth dose of BCG) |  |  |  |  | v | v |  |  |  |  |  |  |
| Vaccination (Birth dose of Hepatitis B) |  |  |  |  | v | v |  |  |  |  |  |  |
| Mosquito net used regularly |  |  |  |  | v | v |  |  |  |  |  |  |
| Exclusive breastfeeding |  |  |  |  | v | v |  |  |  |  |  |  |
| Routine check-up |  |  |  |  | v | v |  |  |  |  |  |  |
| Routine check-up |  |  |  |  | v | v |  |  |  |  |  |  |
| Danger signs surveillance |  |  |  |  | v | v |  |  |  |  |  |  |
| Keeping the baby warm |  |  |  |  | v | v |  |  |  |  |  |  |
| Handwashing before touching the baby |  |  |  |  | v | v |  |  |  |  |  |  |
| Clean safe home & play things |  |  |  |  | v | v |  |  |  |  |  |  |
| Partner/Family participation |  |  |  |  | v | v |  |  |  |  |  |  |
| OPV Vaccination completed |  |  |  |  |  | v | v | v | v | v | v | v |
| Pentavalent Vaccination completed |  |  |  |  |  | v | v | v | v | v | v | v |
| PCV Vaccination completed |  |  |  |  |  | v | v | v | v | v | v | v |
| IPV Vaccination completed |  |  |  |  |  | v | v | v | v | v | v | v |
| MMR Vaccination completed |  |  |  |  |  | v | v | v | v | v | v | v |
| Growth monitoring |  |  |  |  |  | v | v | v | v | v | v | v |
| Continued breastfeeding |  |  |  |  |  | v | v | v | v | v | v | v |
| Mosquito net used regularly |  |  |  |  |  | v | v | v | v | v | v | v |
| Play and communication |  |  |  |  |  | v | v | v | v | v | v | v |
| Birth Registration |  |  |  |  |  | v | v | v | v | v | v | v |
| Diet diversity |  |  |  |  |  | v | v | v | v | v | v | v |
| Adequate meal frequency for age |  |  |  |  |  | v | v | v | v | v | v | v |
| Complementary feeding |  |  |  |  |  | v | v | v | v | v | v | v |
| Vitamin A |  |  |  |  |  | v | v | v | v | v | v | v |
| MUAC |  |  |  |  |  | v | v | v | v | v | v | v |
| Safe drinking water |  |  |  |  |  | v | v | v | v | v | v | v |
| Deworming |  |  |  |  |  | v | v | v | v | v | v | v |
| Partner/family participation |  |  |  |  |  | v | v | v | v | v | v | v |
